# Spencer Technique of the Hip Demonstrates Equivocal Changes in Hip Range of Motion

**DOI:** 10.7759/cureus.83560

**Published:** 2025-05-06

**Authors:** Jesse O'Rorke, Zachary J Buchman, Vincent R Savarino, Jason S DeFrancisis, Cameron S Guay, Brianna Bushman, Benjamin M Vinarski, James Toldi, Rebecca E Steiner, David Boesler

**Affiliations:** 1 Osteopathic Medicine, Lee Health, Fort Myers, USA; 2 Osteopathic Medicine, Lake Erie College of Osteopathic Medicine, Bradenton, USA; 3 School of Medicine, Lake Erie College of Osteopathic Medicine, Bradenton, Florida, USA; 4 Internal Medicine, Lake Erie College of Osteopathic Medicine, Bradenton, USA; 5 Orthopedic Surgery, Lake Erie College of Osteopathic Medicine, Bradenton, USA; 6 Medicine, Lake Erie College of Osteopathic Medicine, Bradenton, USA; 7 Medicine, Lake Erie College of Osteopathic Medicine, Miami, USA; 8 Internal Medicine, Lake Erie College of Osteopathic Medicine, Miami, USA; 9 Sports Medicine, Lincoln Memorial University DeBusk College of Osteopathic Medicine, Orange Park, USA

**Keywords:** athletic performance, omt in sport, orthopedic sports medicine, osteopathic manipulative medicine, spencer technique of the hip

## Abstract

Context

Hip range of motion plays a crucial role in the functional health of runners. Osteopathic manipulative treatment has historically been used to maintain or restore a joint's range of motion; therefore, this study investigates how the Spencer technique impacts femoroacetabular active range of motion in runners.

Objective

This study investigated the impact that the bilateral Spencer technique has on all planes of hip motion.

Methods

Forty participants were randomly divided into treatment and control groups, with the treatment group receiving twice-weekly bilateral Spencer technique for four weeks and the control group receiving no treatment. Both groups were instructed to train as they normally would for the four weeks leading up to a five-kilometer (5K) race. Measurements of hip flexion, extension, internal rotation, external rotation, abduction, and adduction were taken to assess the percent change in range of motion over the course of training, as well as before and after the race, for all planes of motion in treatment vs control groups.

Results

The only significant difference in average percent change in range of motion for control (n=19) vs treatment groups (n=20) across both treatment and race periods was found for abduction wherein the control group experienced an average change of +7.09% (standard deviation of 17.38%) and the treatment group experienced an average change of -5.58% (standard deviation of 17.48%) for a difference of 12.67% (p=0.03). All other findings for all other motions across both training and race periods showed no statistically significant difference (p>0.05).

Conclusion

This study found that four weeks of twice-weekly treatment with the Spencer technique of the hip had no statistically significant impact on change in hip flexion, extension, internal rotation, external rotation, abduction, and adduction across the four weeks when compared to control. When analyzing the impact of this treatment on the change of these motions across a 5K race occurring at the end of the four weeks, this study found that the Spencer technique of the hip had a statistically significant negative impact on abduction, compared to the control. These findings bring into question the role that the Spencer technique of the hip may have for healthy individuals training for athletic competition and open the door for future research to analyze the effect that this osteopathic technique has on motion in a setting that better controls for differences in training habits.

## Introduction

Running is a popular form of exercise enjoyed by millions of people worldwide and is considered the most popular sports activity in several countries. In the United States, about 50 million people participated in running and jogging in 2021 [1]. Being that it is such a common exercise, acute and chronic injuries commonly occur. In fact, according to Yale Medicine, "at least 50 percent of regular runners" report an injury each year [2].

Injuries caused by running can range from acute trauma to chronic overuse injuries and can span across multiple locations. One common location for pain and injury is the hip and groin. Some studies estimate that these injuries account for 5% to 6% of all sports injuries [3]. Additionally, self-reported weak hip muscles and restricted hip mobility may account for even more injuries, even to joints located more distally. For example, in a study that tested hip strength in patients who had knee surgery, they found that there was significant weakness of the hip flexors, extensors, adductors, and abductors compared to the uninjured side (n=34) [4].

To prevent pain and injury in the hip or groin and to improve running performance, increasing hip range of motion may be desired. A study conducted with elite soccer players demonstrated this by finding that those with a lower preseason hip flexor range of motion had a higher risk of muscle strain injury during the season (n=36) [5]. Therefore, to improve hip range of motion, osteopathic manipulative therapy (OMT) in the form of the Spencer technique was investigated to achieve this goal. This technique is done by bringing the joint to its restrictive barrier and applying an articulatory technique, which involves using rhythmic, repetitive, direct, and indirect movements through the hip's range of motion. This is done in eight steps in the following order: flexion, extension, circumduction with traction and compression, internal rotation, external rotation, abduction, and adduction [6]. This therapy has been well studied in the shoulder and has been shown to increase range of motion in multiple planes in patients with adhesive capsulitis [7]. However, there is a significant lack of research surrounding its effect on the hip. In fact, there is no study done demonstrating its effect on the hip, let alone in runners. To fill this gap, we investigated the effect of the Spencer technique on hip range of motion in runners.

## Materials and methods

To evaluate the impact of the Spencer technique on hip joint range of motion and femoral-acetabular mobility, an Institutional Review Board (IRB) approval was requested for this study. The IRB proposal was approved and assigned number 31-085. A post hoc clinical trial registry was made through ClinicalTrials.gov and was assigned as number NCT06644989. Study funding was provided through LECOM LCAE grant #J2023.23, and $2550.00 was appropriated to finance the project. Informed consent was obtained by all participants, which occurred prior to the start of the study and after the initial screening requirements. Informed consent was completed on a paper form explaining the summary of information, the purpose of the study, inclusion and exclusion criteria as displayed in Table 1, procedures, potential risks, potential benefits, confidentiality, voluntary participation, withdrawal from participation, involuntary withdrawal and an opportunity for prospective participants to ask questions. Signatures of all participants and investigators were obtained. Participants were compensated through an Amazon.com gift card worth $20.00 as well as coverage of their LECOM 5K registration fee worth $40.00.

**Table 1:** All of the inclusion and exclusion criteria used in participant recruitment and enrollment In order to be successfully enrolled, the potential participant had to agree to complete, or be in compliance with, all the inclusion criteria and none of the exclusion criteria.

Criteria type	Criteria
Inclusion criteria	1. Be a current 1st or 2nd year medical student at LECOM-Bradenton, between ages 21 and 30
	2. Attend all three measurement sessions
	3. If in treatment group, attend 100% of treatment sessions
	4. Participate in one 5-kilometer race, in a group
Exclusion Criteria	1. If they cannot sign an informed consent
	2. If they are pregnant or plan to become pregnant
	3. If they have a past medical history which increases the risk of adverse reactions including, but not limited to, any of the following conditions: skin disorders or open wounds precluding skin contact, neurological symptoms (i.e. numbness, tingling, weakness), adhesive capsulitis of the hip joint, osteoarthritis of the lower extremity, rheumatoid arthritis, gout, iliotibial band syndrome, Legg-Calve-Perthes disease, slipped capital femoral epiphysis, hip dysplasia, avascular necrosis of the hip, chronic hip bursitis, hip dislocation, osteoporosis/osteopenia, severe femoroacetabular impingement, immunosuppressive syndromes, radiation or chemotherapy within the past 3 years, congestive heart failure, Down syndrome, have been told by a physician or other medical professional that you should not participate in aerobic training activities, or have medication changes in the last month
	4. If they have a past medical history which increases confounding variables to the study including, but not limited to, any of the following conditions: recent bone fracture, use of heel lift due to leg length discrepancy, recent lower extremity ligamentous sprain, recent lower extremity muscle strain, history of hip or knee arthroplasty, or 1 year history of any lower extremity surgery
	5. Additionally, participants will be excluded if they report that they run more than 30 miles per week on average

To test the precision of the goniometer measurements prior to study onset, preliminary data was collected by each member of the research team, measuring all planes of motion on six standardized individuals. The measurements for each motion, on each individual, were compiled to generate an average standard deviation for each motion, for which results can be found in Table 2 in the results section. These results were used to help us establish inter-measurer reliability to ensure there were no outliers within the student researchers.

**Table 2:** The protocol of the Spencer technique of the hip which was used in this study Each step was performed bilaterally and as adherent to the directions provided in the Atlas of Osteopathic Techniques, as possible, with standardized procedures. Standardized procedures were required to be implemented as there is variation in terms of the number of thrusts per stage, which is acceptable, number of circumductions in stages three and four. For all stages, the patient is in a supine position, and each step was done one time per leg at each treatment session ASIS - anterior superior iliac spine

Stages	Steps
Stage 1: hip flexion	1) The physician stands next to the hip being treated.
	2) The physician bends the knee and carries the hip to the flexion-restrictive barrier, and an articulatory motion is applied at the end range of motion
Stage 2: hip extension	1) The physician stands next to the hip being treated
	2) The patient's leg is moved off the side of the table slightly and depressed at the distal thigh until it meets its extension-restrictive barrier
	3) The physician stabilizes the opposite ASIS, and an articulatory motion is applied at the end range of motion
Stage 3: Circumduction with compression	1) The physician stands next to the hip being treated
	2) The patient's leg is moved off the side of the table slightly and depressed at the distal thigh until it meets its extension-restrictive barrier
	3) The physician stabilizes the opposite ASIS, and an articulatory motion is applied at the end range of motion
Stage 4: circumduction with traction	1) The physician, standing at the foot of the bed
	2) The physician extends the patient’s knee and grasps the ankle, adding moderate traction
	3) Continuing to hold traction, the physician circumducts the patient’s hip through five small and enlarging circles (clockwise, then counterclockwise directions) while maintaining traction
Stage 5: internal rotation	1) The physician stands opposite to the hip being treated
	2) The physician flexes the patient's hip and knee and internally rotates the hip to its barrier while stabilizing the ipsilateral ASIS to the side being treated, and an articulatory motion is applied at the end range of motion
Stage 6: external rotation	1) The physician stands opposite to the hip being treated
	2) The physician flexes the patient’s hip and knee and externally rotates the hip to its barrier while stabilizing the contralateral ASIS to the side being treated, and an articulatory motion is applied at the end range of motion
Stage 7: abduction	1) The physician stands next to the hip being treated
	2) The physician takes the patient’s straightened leg and abducts it to its restrictive barrier. The physician stabilizes the ipsilateral ASIS, and an articulatory motion is applied at the end range of motion
Stage 8: adduction	1) The physician stands next to the hip being treated
	2) The patient’s leg is slightly elevated with the knee extended
	3) The hip is taken to the adduction barrier while maintaining stabilization of ipsilateral ASIS, and an articulatory motion is applied at the end range of motion.

**Figure 1:**
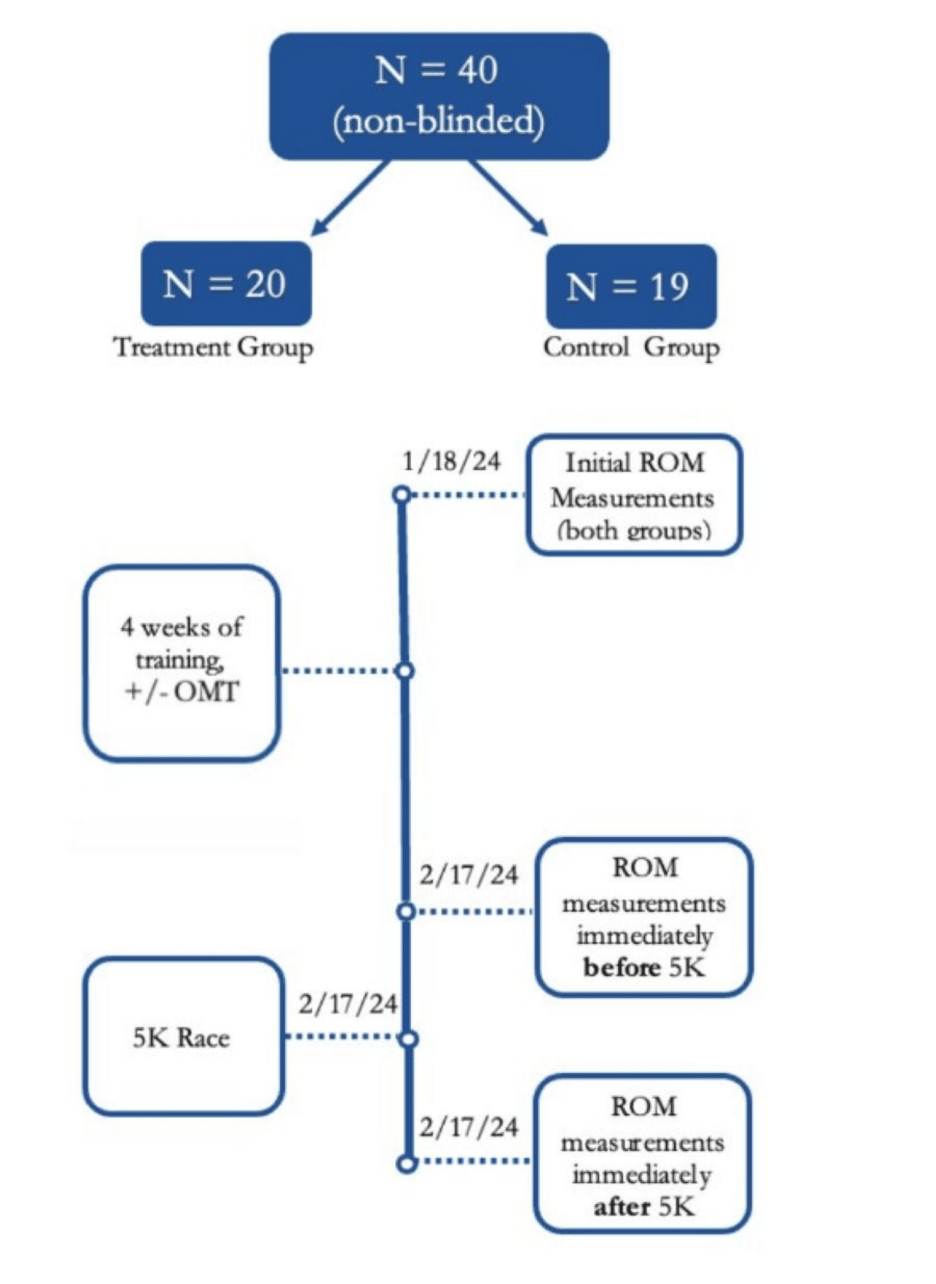
Timeline of study with associated dates that involved events were completed Original enrollment occurred prior to initial ROM measurements and the last three events, all regarding the race, occurred on the same day, February 17, 2024. ROM - range of motion; OMT - osteopathic manipulative therapy

Participants of the study consisted of first- and second-year medical students who were recruited via email. Interested medical students responded to the email, and potential participants were screened according to the inclusion and exclusion criteria (Figure 1). Those eligible to participate in the study were selected. Participant selection was done in a consecutive manner, and 40 participants agreed and signed consent forms, officially enrolling them in the study. 

After the participants were officially enrolled, all participants attended a session where their initial hip range of motion (ROM) measurements-encompassing flexion, extension, abduction, adduction, internal rotation, and external rotation-were recorded in degrees for each participant using a goniometer, where the measurers were blinded to which participants were in the treatment vs. control group. After initial hip ROM measurements were taken on the study participants, 40 participants were randomly assigned to the treatment group (n=20) or the control group (n=20). Participants in both groups were instructed to train as they normally would, knowing they were running a 5K race in the near future. The treatment group received the Spencer technique of the hip, bilaterally, twice weekly for four weeks. All members of the research team who are medical students participated in providing treatment to the study subjects. To ensure proficiency, prior to treatment, all authors exhibited capability through a series of demonstrations of the Spencer technique, in accordance with the direction of the Atlas of Osteopathic Techniques [6]. An experienced osteopathic physician oversaw and confirmed their proficiency via two consecutive successful demonstrations. In order to ensure equal treatment was provided to participants of the treatment group, the Spencer technique was standardized and performed as demonstrated in Table 2*.* The control group received no treatment for four weeks.

Following the four weeks, two sets of ROM measurements were taken on the day of the race, two days following the last Spencer treatment for the treatment group. A set of pre-race ROM measurements and post-race ROM measurements was obtained. All data was collected, and the authors proceeded with the analysis of the data. A summary of the timeline of this study is displayed in Figure 1.

For the three times data were collected, the degrees of flexion (F), extension (E), internal rotation (IR), external rotation (ER), abduction (AB), and adduction (AD) were measured bilaterally. These bilateral measurements were subsequently averaged. This resulted in final data of average range of motion (ROM) for each movement measured at three distinct points in time: 1) initial measurements (Jan 18, 2024 5:00 PM); 2) pre-race measurements (Feb 17, 2024 7:00 AM); and 3) post-race measurements (Feb 17, 2024 9:00 AM).

This allowed for the determination of the percent change of each motion between each point in time. This data was compared between treatment and control groups, across two non-overlapping time periods: 1) training period, which is designated as the time between initial measurements and pre-race measurements; and 2) race period, which is designated as the time between pre-race and post-race measurements

Analysis of these data (percent change in ROM in control group vs treatment group for both the training and race periods) was performed using two-sample, two-tailed T-tests.

## Results

In regards to inter-researcher precision of goniometric measurements, Table 3 demonstrates the findings for average standard deviations for each motion for measurements done by all members of the research team on standardized individuals, prior to the study's onset. There was greatest variability in terms of inter-researcher data collection for abduction (average standard deviation of 7.72 degrees) and least variability for extension (average standard deviation of 4.00 degrees). Further, there were zero outliers (defined as ± 3 standard deviations from the mean) in terms of measurements taken by members of the research team on the standardized individuals.

**Table 3:** Findings of average range of motion and standard deviation, in degrees, for each motion taken by members of the research team on six standardized individuals

Motion	Average degrees range of motion	Average standard deviation (degrees of motion)
Flexion	100.05	6.1
Extension	13.98	4.0
Internal rotation	27.69	5.2
External rotation	23.05	5.0
Abduction	33.05	7.7
Adduction	23.62	5.8

A total of 39 participants successfully completed all the requirements of the study (including running the five-kilometer race and attending all measurement sessions), with only one of the original 40 participants being lost during the four weeks. This individual was a member of the control group, leaving 19 participants in the control group and 20 participants in the treatment group. The participants in the treatment group had a 100% attendance rate for their twice-weekly treatment sessions.

The results of the analyses comparing average percent change in ROM for control vs treatment group across both the training and race periods can be seen in Table 4 and Table 5. The only significant difference in average percent change in ROM for control (n=19) vs treatment groups (n= 20) across both treatment and race periods was found for abduction wherein the control group experienced an average change of +7.09% (standard deviation of 17.38%) and the treatment group experienced an average change of -5.58% (standard deviation of 17.48%) for a difference of 12.67% (p=0.03). All other findings for all other motions across both time periods showed no statistically significant difference (p>0.05).

**Table 4:** All findings regarding control vs treatment average percent change in ROM across training period ROM - range of motion

Motion	Average % change in ROM (control Group)	Average % change in ROM (treatment group)	Difference (%)	p-value
Flexion	+4.5% (±9.9)	+2.0% (±9.9)	2.5	0.4
Extension	+11.2% (±34.0)	+18.6% (±39.7)	7.4	0.5
Internal rotation	+23.4% (±20.2)	+9.5% (±31.1)	13.9	0.1
External rotation	+9.0% (±32.1)	+11.7% (±30.8)	2.7	0.7
Abduction	+14.9% (±19.3)	+29.5% (±37.0)	14.6	0.1
Adduction	+14.6% (±43.2)	+15.5% (±39.9)	0.9	0.9

**Table 5:** All findings regarding control vs treatment average percent change in ROM across race period ROM - range of motion

Motion	Average % change in ROM (control group)	Average % change in ROM (treatment group)	Difference (%)	p-value
Flexion	-3.4% (±8.29)	-2.8% (±7.77)	0.6	0.8
Extension	-0.5% (±25.7)	-1.6% (±31.6)	1.1	0.9
Internal rotation	+7.4% (±22.59)	+10.5% (±28.6)	3.1	0.7
External rotation	+25.5% (±34.8)	+12.8% (±29.9)	12.6	0.2
Abduction	+7.1% (±17.3)	-5.6% (±17.5)	12.7	0.03*
Adduction	+5.6% (±29.5)	+9.6% (±40.3)	4.0	0.7

## Discussion

Our findings demonstrated that during the four weeks leading up to the 5K, the Spencer technique had no statistically significant impact on any plane of hip motion compared to control. Additionally, those who received the Spencer technique experienced a slight decrease in abduction over the course of the race, whereas those in the control group experienced a slight increase in this same motion, a statistically significant difference. A wide variety of factors may have contributed to these findings, which fail to demonstrate a beneficial impact that the Spencer technique has on a runner's range of motion. First, it is possible that four weeks of twice-weekly treatment was simply not enough in terms of quantity of treatment to see a significant benefit of the technique, as multiple days in between the treatments could, in theory, impair a compounding beneficial effect. It is also possible that the findings of this study were due to differences in training habits, as there was no specific training guideline given to the participants, leaving room for differences in how individuals trained for the race. Further, this does not need to simply refer to aerobic or anaerobic training either, as differences in stretching habits could certainly impact these data, as could unreported injuries since training was done unsupervised. Not having a standardized training program is one of the greatest challenges of this study, however, this leaves a great opportunity for team-focused research in the future, since in this setting, training regimens could be more identical. From the perspective of this research, however, the goal was set to investigate the technique without intervening with training patterns so that the results could be generalized to a wider population.

Another factor impacting the internal validity is the fact that the measurements used in this study were done via a hand goniometer by members of the research team. Although the process was standardized and all members of the research group received equal training, producing data demonstrating adequate inter-measurer reliability, advances in video-based goniometry could have enhanced the accuracy and precision of measurements taken for this study [8]. The main factor affecting external validity is the limited age range from which the participants were selected. Each participant in the study was a pre-clinical medical student (between the ages of 21 and 30) without any major health concerns, thus excluding adolescents, the elderly, and sick or injured adults from the study. Using a healthy pool of participants being treated with a technique designed to remedy somatic dysfunction may be ultimately why the findings were produced as such. 

Osteopathic physicians who specialize in sports medicine, physical medicine and rehabilitation, or neuromusculoskeletal medicine may find great utility in treating patients with this technique who come in with ailments limiting their hip range of motion. This study has the opportunity to be reproduced beyond the limited age range and sample size that were present in order to further determine the clinical impact of the findings produced. 

Prior research related to the Spencer technique of the shoulder incorporated a baseball team to determine the impact on range of motion in these athletes [9]. Additionally, a study of twenty nine elderly patients with "common shoulder problems" was evaluated after 14 weeks in which they received five Spencer technique of the shoulder treatments and was shown to have a statistically significant increase in range of motion and a subjective decrease in perceived pain compared to a placebo group [10]. Similarly, applying this study's methodology to an organized track and field team could help to increase the internal validity by implementing standardized activity levels for all members of the team based on training regimens.

Aside from standardization of treatment protocols, it would be of great use to clearly define the target patient population, to include diagnosed musculoskeletal conditions, severity of conditions, and present comorbidities, to evaluate present confounders that may impact the benefit of the Spencer technique. Additionally, evaluating treatment results by including subjective measures such as pain scale and patient quality of life would be a useful comparison to compare the Spencer technique to placebo exercises in order to examine an unintended benefit of the study, patient satisfaction [11]. The introduction of the Spencer technique in multiple locations with trained providers can help to produce greater generalizability and further support the use of this technique for the treatment of musculoskeletal conditions in the future.

Future studies should focus on how the Spencer technique of the hip impacts those of varying activity levels and ages while also standardizing outside factors such as activity level and stretching habits.

## Conclusions

This study found that four weeks of twice-weekly treatment with the Spencer technique of the hip had no statistically significant impact on change in hip flexion, extension, internal rotation, external rotation, abduction, and adduction across the four weeks when compared to control. When analyzing the impact of this treatment on the change of these motions across a 5K race occurring at the end of the four weeks, this study found that the Spencer technique of the hip had a statistically significant negative impact on abduction, compared to the control. The methodology of this study did not account for differences in training habits in the hope of being able to externalize these findings more greatly, leaving room in the future to expand research with this in mind. Also, using a participant pool of young, healthy adults without any history of diagnosed somatic dysfunctions limits the external validity in that many of those who deal with ailments related to reduced hip range of motion do not fall within this demographic. Ultimately, these findings bring into question the role that the Spencer technique of the hip can have for healthy individuals training for athletic competition and opens the door for future research to analyze the effect this osteopathic technique has on hip range of motion in a setting that better controls for differences in physical training habits.
